# Computational investigation of inhibitory mechanism of flavonoids as bovine serum albumin anti-glycation agents

**DOI:** 10.1186/s40199-014-0079-0

**Published:** 2014-12-11

**Authors:** Anahita Johari, Ali Akbar Moosavi-Movahedi, Massoud Amanlou

**Affiliations:** Department of Medicinal Chemistry, Faculty of Pharmacy and Pharmaceutical Sciences Research Center, Tehran University of Medical Sciences, Tehran, Iran; Institute of Biochemistry and Biophysics, University of Tehran, Tehran, Iran

**Keywords:** Homology modeling, Molecular dynamics simulation, Correlation analyzes, Glycation sites, Flavonoids, BSA

## Abstract

**Background:**

Glycation of serum albumin and its consequence products were considered as an important factor in drug distribution and diabetic complications, therefore finding the glycation inhibitors and their inhibitory mechanisms became a valuable field of study. In this work, bovine serum albumin (BSA) became a subject as a model protein for analyzing the inhibitory mechanism of flavonoids, known as natural BSA glycation inhibitors in the early stage of glycation.

**Methods:**

Firstly, for theoretical study, the three-dimensional model of BSA structure was generated by homology modeling and refined through molecular dynamic simulation. Secondly, several validation methods (statistical assessment methods and also neural network methods) by simultaneous docking study were employed for insurance about accuracy of our simulation. Then docking studies were performed for visualizing the relation between flavonoids’ binding sites and BSA glycation sites besides, the correlation analyzes between calculated binding energy and reported experimental inhibitory IC_50_ values of the flavonoids set, was considered to explore their molecular inhibitory mechanism.

**Results:**

The quality assessment methods and simultaneous docking studies on interaction of quercetin (as the most studied flavonoids) with BSA and Human serum albumin (HAS), confirm the accuracy of simulation and the second stage of docking results which were in close agreement with experimental observations, suggest that the potential residues in flavonoids binding sites (which were place neighbor of tryptophan 212 within 5Ǻ) cannot be considered as one of glycation sites.

**Conclusions:**

Based on the results, flavonoids don’t participate in inhibitory interference mechanism, and also, the differentiation between complexes of flavonoids with BSA and HSA could destroy the speculation of using them as an exchangeable model protein in study of serum albumin and flavonoids interactions.

## Background

Diabetes is considered as one of the main threat to human health, according to WHO report, it is predicted that diabetes will exceed to 300 million in the years of 2025. Diabetes has a devastating effect on almost every organ in body. The hyperglycemia in diabetic conditions causes non-enzymatic glycation of proteins [1]. This reaction initiates the formation of reversible Schiff base between glucose and ε-amino group of lysine residues in proteins which by consequent intermolecular rearrangements leading to a formation of ketamine adduct. Then, the potent precursors of protein cross-linkers such as glucosone, 5-hydroxymethyl-2-furaldehyde, and 3-deoxyglucosone are formed due to the initial reaction. Next, those intermediates are oxidized, and cross linked, and fluorescent heterogeneous group of protein-bound moieties (called advanced glycated end products or AGEs) are detected in the last stage of glycation [2,3]. With body of evidence, formation and accumulation of advanced glycation products can be considered as a major factor in development of diabetic complication, Alzheimer’s disease, and normal aging process. Therefore, the considerable efforts have been made to propose and discover inhibitors of glycation for therapeutic application in treatment of diseases that advanced glycation end product could be responsible for their pathogenesis [4].

Serum albumin is the most abundant protein in body which has a tremendous interest for binding to various natural or synthetic small-molecules such as glucose and related compounds. In diabetic condition, serum albumin undergoes glycation [5]. This character, continually expose serum albumin to altering factors which can participate in serum albumin modifications and biological compound formation that can be considered as a causative agent for some diseases [6]. Among the wide variety of interaction, the none-enzymatic interaction of serum albumin with glucose is one of the most important underlying modification factors, which are in associated with various alterations of albumin’s structure and functions [7,8].

Among the glycation inhibitors’ which were examined on bovine serum albumin (BSA) as a model protein, specific considerations were given to dietary natural products such as several phenolic compounds [9,10]. Researches on a limited number of flavonoids compounds indicated their ability to prevention both stage of glycation reaction [7]. In the late stage, the inhibition mechanisms of flavonoids are that, they may act as a both metal chelator and radical scavenger [11,12]. However, the inhibitory mechanism of flavonoids in the early stage completely remains unclear [7]. For some anti-glycation agent such as α-lipoate or non-steroidal anti-inflammatory drugs such as diclofenac and aspirin, hydrophobic hindrance or binding near the glycation site was considered as possible mechanism of anti-glycation activity [9,13]. Although we have no evidence that flavonoids react with the glycation site [7], in the present study, the attentions are drawn to binding behavior of flavonoids to clarifying the inhibition mechanism of them for the early stage of glycation.

Since, docking simulation results can be used to understanding the molecular interaction and analyze biological presses, rapidly and efficiently [2], in the present study, we explored the series of flavonoids’ binding behavior with BSA through docking studies.

However, in the case of BSA which was considered here as a model protein (such as studies mentioned above by Morimitsu, Y., et al. and Matsuda, H., et al. [3,12]). When this study was started nobody has succeeded in obtaining crystals of intact BSA [14]. However, simultaneous with our study the importance of this protein draws the other researchers’ attention to solving the tertiary structure of BSA. However, the new crystals also haven’t been at high resolution (<2 Å resolution limit) [15], which can be satisfactory resolution for using in docking simulation. In this study, simulation, homology modeling was employed. While, it is generally recognized that homology modeling of proteins could be considered as accurate methods for 3D structure prediction while a model and related template sharing high sequence identity [16,17].

In present work, the model was refined with energy minimization and molecular dynamic simulation. Then, different assessment tools were adapted for checking the proper folding of constructed BSA 3D structure.

Consequently, by docking methods the validity of the model protein was examined and for getting better insight into the flavonoids’ inhibitory mechanism. Firstly, the predicted binding affinity and reported IC_50_ [12] was subjected to correlation analyzes and secondly, the flavonoids binding location was studied for tracing the relation among participated amino acids in the flavonoids’ binding site with ones proposed as the glycation site of BSA [18].

In addition, according to docking results, the comparison is made between obtained 3D structure of BSA and crystallographic structure of HSA for understanding their specificity in flavonoids binding location and the verification of using BSA and HSA as an exchangeable mode protein for the same case of investigation.

## Methods

### Amino acid sequence alignment and homology modeling of bovine serum albumin

In the first step, Amino acid sequence of BSA (UniProt ID: P02769) was retrieved from Swiss-Prot (http://www.uniprot.org/uniprot). And then, Blastp matrix blosum62 against PDB database was used to find out the best homologous structure with a known 3D structure, for applying as a template in homology modeling. Finally, among the HSA structures, the structure with a highest resolution at 1.9 Å [19] that belong to HSA (PDB ID: 1N5U) was adopted as the final target.

Sequence alignment was generated using 2d alignment function of MODELLER 9v8 program (www.salilab.org/modeller). Align 2d is based on a dynamic programming algorithm, considering the template structural information for constructing an alignment, this advantages is more significant as the similarity between the sequences decrease and the number of gaps increase [20]. After alignment, the 3D structure of fifty models were calculated completely automatically with MODELLER 9v8 [21].

### Refinement of homology model by minimization and MD simulation

Molecular dynamic simulation were carried out using the parallel version of GROMACS 4.5.3 [22] In the AMBER99SB force field [23] to monitor stability and conformation of the selected model. The starting coordinates were taken from the best model that was retrieved from MODELLER and for prediction of non-standard ionization state of residues PROPKA 2.0 was applied [24]. Among the whole residues that may be adapted standard ionization state, lysine 219 was deprotonated and six histidine residues (histidine 16, histidine 37, histidine 143, histidine 239, histidine 335, and histidine 332) were completely protonated.

After assigning charge state, the protein was put into a suitable sized simulation cubic box and immersed in 41760 simple point charges (SPC) water model. Then, appropriate number of Na^+^ ions were added to neutralize the charge of the system. The first energy minimization, which targeted water molecules and ions alone while configuration of protein was kept, fixed, followed by 20 ps MD simulation which again, the protein was kept fixed and the water molecules and ions were allowed to evolve. The second minimization step began with 1000 steepest descent and conjugate gradient of 9000 steps afterward. For equilibrating further the system v-rescale thermostat [25] was applied by using coupling constant (τ_t_) of 0.1 ps to keep the system at the constant temperature in 100 ps at 100 K and 200 K respectively. While the pressure of the system maintained at 1 atm by using the Berendsen weak coupling algorithm [26] with coupling constant (τ_p_) of 0.5 ps. For obtaining the stable system at 300 K and 1 atm, the same aforementioned parameters were determined for heating the system.

In this simulation, the non-bonded interactions were calculated by applying a 14 Å cut off and periodic boundary conditions were also applied in all directions. The Electrostatic interactions were calculated using the Particle Mesh Ewald (PME) [27]. During simulation, the frame was stored at every 1.000 ps.

Subsequently, all the analysis was performed by taking advantage of Gromacs tools and the structure which was retrieved after system stabilization was subject to evaluate the protein geometry.

### Assessment of the homology model

To verify the quality of the model after molecular dynamic refinement, using different tools, which include: PRoQ, as a neural network based predictor [28], verify 3D, which analyzes the compatibility of an atomic model (3D) with its own amino acid sequence (1D) [29], PROSA program, which evaluate the energy of using a distance based pair potential [30] as well PROCHECK for checking the detailed stereochemical properties [31].

### Docking analysis

Autodock 4.0 [32] was used for docking studies and for adding charges and active torsions to the ligands and assign Kollman charges to protein AutoDockTools (ADT) was employed. The rectangular lattice (100 × 100 × 100) with points separated by 0.375 Ǻ was built by AutoGrid that was superposed on subdomin IIA of serum albumin (proposed as a primarily binding site of flavonoids) [33]. The Lamarckian genetic algorithm (LGA) was used with 100 runs for docking simulation, while an initial population size of 100, and the maximum number of 2500000 energy evaluations. All other parameters were set to default values, and the results were ranked according to Autodock scoring function and the final conformation of each structure was selected based on the highest dock score.

The chemical structures of interested flavonoids were optimized at MM+ level of molecular mechanic methods using HYPERCHEM software program (version 8.0.3 Hypercube, Inc) [34]. The geometry optimization was preceded by Polak-Ribiere algorithm to reach the 0.01 root mean square gradient. Then all these refinement structures were subject to docking simulations.

### MD simulation after docking

All MD simulations were performed using the Gromacs 4.5.3 program together with the 43a2 force field [35-37] for each simulation to study the complex of BSA-quercetin, while the topology of quercetin was generated by the PRODRG 2.5 beta server [38].

Then, the protein was solvated by simple point charge water molecule in the cubic box with minimal distance of protein from its wall being 1.0 nm and the system was neutralized by adding appropriate counter ions. After performing energy minimization of the entire system, it was equilibrated under NVT ensemble for 100 ps and the periodic boundary conditions were used in tree-coordinate direction, whereas, position restrains were placed on ligand using force restraint of 1000 kcal/mol. NVT equilibration is followed by isothermal–isobaric (NPT) which was performed for 100 ps. The pressure of the system was regulated by Berendsen barostat with a relaxation time (τp) of 2.0 ps. The position restraints and simulation parameter which was applied in the NPT ensemble was the same as the ones which have been used during NPT equilibration. After convergence had been reached, the protein-ligand and water-ions groups were coupled in separate temperature baths, and v-rescale thermostat was used to keep the temperature constant at 300 k during the 2 ns simulation. Lennard-Jones interactions were calculated within a cut-off distance of 1.0 nm, and electrostatic interactions were treated with the particle mesh Ewald’s method. Ultimately, from 2 ns MD simulation, the structure with low RMSD value was selected for analyzing the local conformational changes of BSA- quercetin complex.

### Computational resources

The computational studies were performed on computer cluster 4 HP Prolient ML370-G5 Tower servers equipped with 2 Quad Core Intel Xeon processors E5355, 2.66 GHz and 4GB of RAM on a Linux platform.

## Results

### Sequence alignment and modeling building

Based on the highest similarity and the lowest D-value, human serum albumin (HSA) can be considered the best homologues structure for BSA. The Sequence alignment of BSA (UniProt ID: P02769) and HSA (UniProt ID: P02768) which was generated by ClustalX program shows 76% identity between them [39]. The precursor proteins of BSA and HSA have 603 and 607 amino acid in length, respectively. Though all the crystallographic structures of HSA which were reported, only consist of 583 amino acids. This can be referred to the fact that during a secretion and maturation process of serum albumins, the 25 N-terminal amino acids were cleaved [40]. Therefore, the 25 N –terminal amino acids, which are placed out of the chain, were separated from the HSA amino acids sequence and the rest of them used as a target for homology modeling.

**Figure 1:**
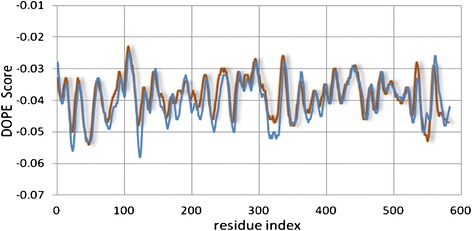
**Analysis of DOPE score profile (obtained from Modeller).** The Blue line indicates, energy profile of HSA (PDB ID: 1N5U) which used as a model and the red line indicates energy profile of target, which constructed by Modeller.

After alignment, the 3D structure of fifty models was calculated completely automatically with modeler 9v8. In this process, the population of the constructed model was increased for improving the DOPE energy assessment and MODELLER objective function calculation and then the best model was selected based on the lowest DOPE score. Next, for gave an idea about the quality of input alignment the DOPE score profile (Figure 1) was calculated with “assess_dope” function of MODELLER. It can be seen in Figure 1, that in the whole range of residues, the DOPE score of both proteins converges and the results aren’t showing any problematic regions.

### Analysis the trajectory of the molecular dynamics simulation

**Figure 2:**
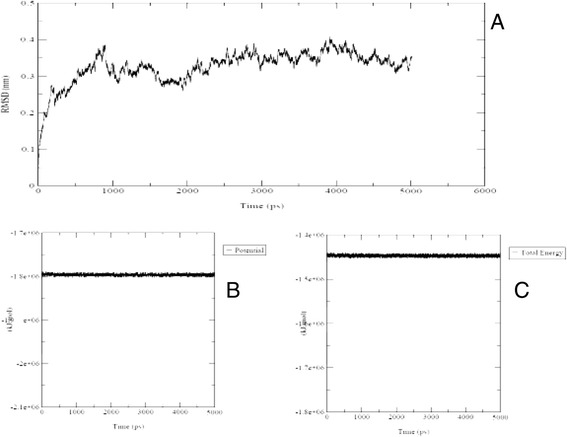
**Analysis of a molecular dynamics simulation trajectory during 5ns generated by Gromacs. (A)** The backbone root mean square deviation (RMSD) plot of the model, during 5ns simulation. **(B)** The variation of potential around constant value. **(C)** The fluctuation of total energy.

During 5 ns MD simulation, the system was analyzed for its stability. The deviation plot of potential, and root mean square deviation (Figure 2) were derived from the respective trajectory by Gromacs software output. The low degree of RMSD fluctuation during last 2 ns indicates that the system tends to be converging and therefore, it is become stable. According to what illustrated in Figure 2, the total energy of the complex was observed within the range from −1.4 to −1.5 kJ/mol, which can be a reasonable value.

### Assess the validity of generated model after MD

In present work two statistical approaches such as verify3D and PROSA confirm a reliability of the constructed model.

**Figure 3:**
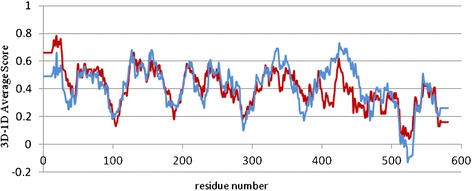
**The Verify-3D analysis is shown for all residues.** The blue line belongs to molecular structure after MD, and red one belongs to model that constructed by the Modeller. The refinement model after MD was validated by positive scores of Verify-3D.

In the first approach, the Verify 3D profile (Figure 3) represents that 3D-1D average score of the most residues in the model proteins both before and after MD, given a score over 0.2 except the ones rage 514–532. In the model protein that refined after MD, the whole residues have a positive score, whereas in the model that constructed by the modeler the amino acids in the range of 518–530 have a negative score. Therefore, based on these results, the sign of model folding improvement after molecular dynamic simulation is traceable.

**Figure 4:**
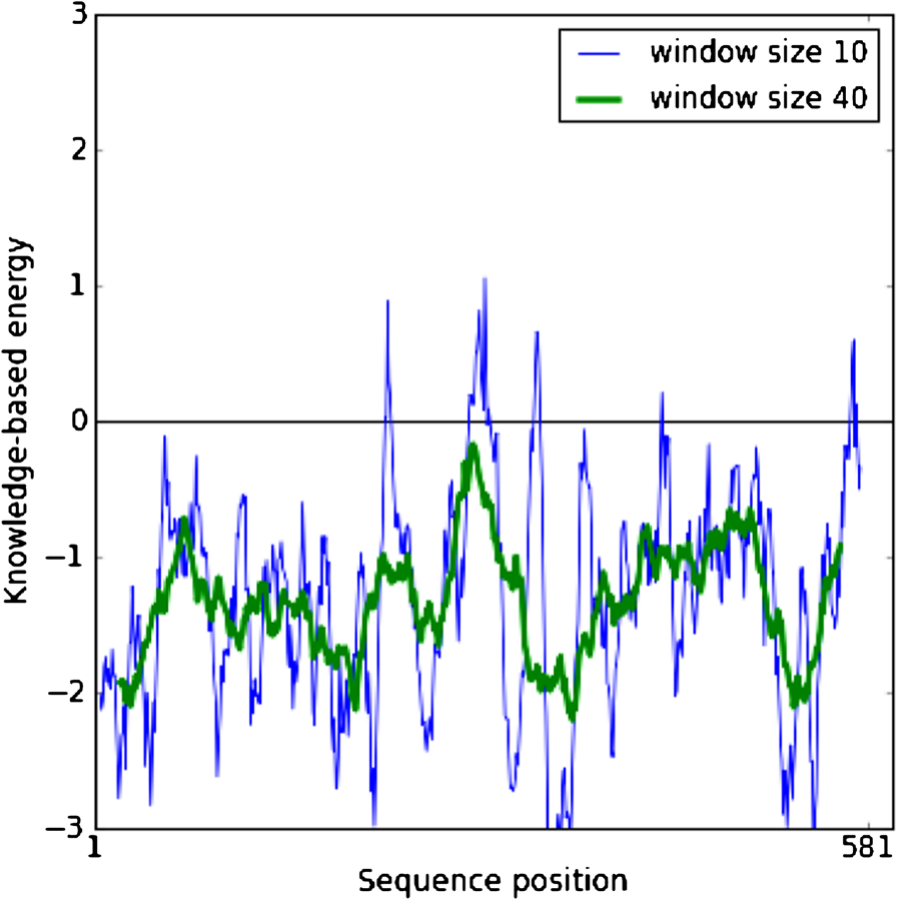
**Energy plot from ProSA program, all residues have negative ProSA energies which confirms the reliability of the model.**

The second statistical assessment method was processed with ProSA program. The Normalized Z-score calculation indicates standard deviation value is found in the range of native conformation of crystallographic structure and also according to Figure 4, ProSA score, which was calculated interaction energy per residues, has a negative score across the most of the residues’ length.

After performing those earlier methodologies based on statistical approaches (Verify3D, ProSA), the PRoQ was applied to compare the validity of the model before and after MD simulation. This program is based on a neural network that by considering the Wallner and Elofsson’s study it is indeed superior to statistics-based methods [41]. In this method two quality measures are predicted, LG score and MaxSub score. Based on their range of qualities; predicted LG score and MaxSub score of the model after MD is 4.14 (which is within the range of a ‘extremely good’) and 0.45( which are within the range of ‘good’), while values of 2.915 and 0.304 were obtained for LG score and MaxSub score, respectively in the model before MD simulation. These scores indicate the refinement of the model after MD simulation.

**Figure 5:**
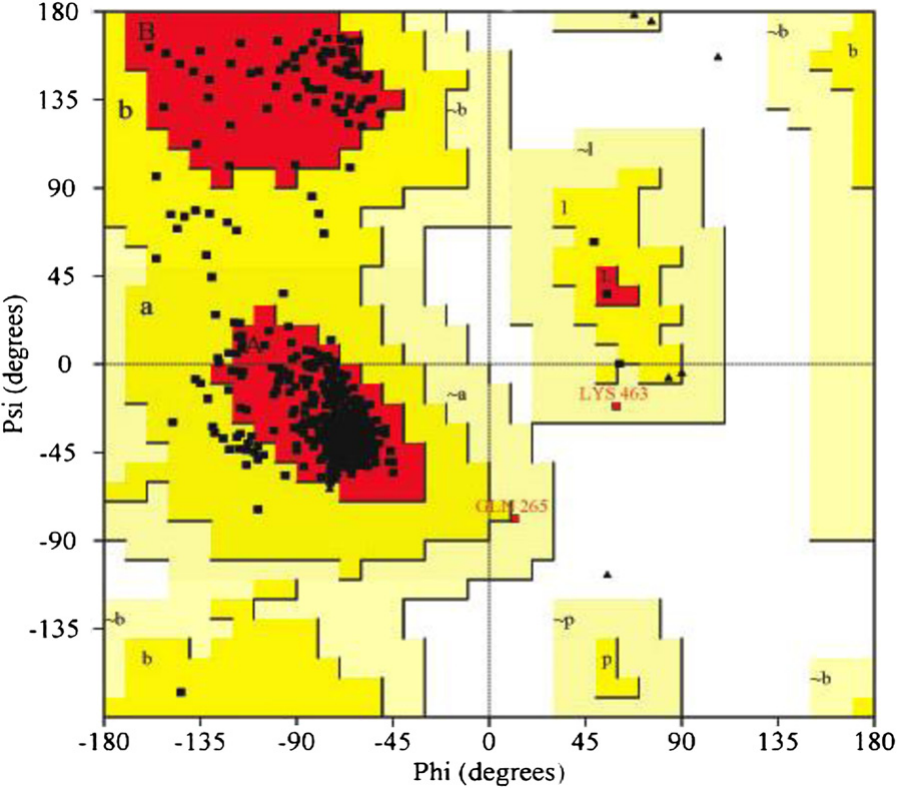
**Ramachandran plot for 3D structural model of bovine serum albumin.**

Ultimately, the validity of the model was evaluated by PROCHECK, and stereochemical properties of protein structure were listed in Tables 1 and 2 [31,42]. In the Figure 5, phi and psi distribution of Ramachandran plot indicate that 100% of residues were present in favored and allowed regions, which confirm that the structure is comparable with good homology model [43]. Also the main chain parameters which consist of six properties plotted are all well within the established limit of reliable structures (Table 2).

**Table 1:** **Ramachandran plot statistics**

% residues in most favored regions	90.8%
% residues in additional allowed regions	8.8%
% residues in generously allowed regions	0.4%
% residues in disallowed regions	0.0%
% Num. of non-glycine and non-proline residues	100.0%

**Table 2:** **Main chain parameters plot statistics**

**Stereochemical parameter**	**No. of data pts**	**Parameter value**	**Comparison values**	**Comparison values**	**No. of band widths from mean**	
			**Typical value**	**Band width**		
%-tage residues in A,B,L	535	90.8	84.9	10.0	0.6	Inside
Omega angle Std. dev.	380	7.0	6.0	3.0	0.3	Inside
Bad contacts/100 residues	0	0.0	3.3	10.0	−0.3	Inside
Zeta angle Std. dev.	565	1.3	3.1	1.6	−1.1	Better
H-bond energy Std. dev.	400	0.7	0.8	0.2	0.5	Inside
Overall G-factor	581	0.0	−0.3	0.3	1.0	Better

In brief, according to all applied evaluated methods, it can be concluded that obtained 3.D structural model has an acceptable geometry for further study by docking approach.

### Docking studies

With the interest of comparability of docking result with experimental data (which studied the interaction among flavonoids and BSA), and also shedding the light at an ambiguous point of experimental observation in molecular level, with respect to Dufour and Dangles work [33] one stage of docking simulation was designed. Therefore, the binding interaction of quercetin (as the most studied flavonoid) and warfarin (as a site marker of subdomain IIA HSA) was subject to docking simulation.

**Figure 6:**
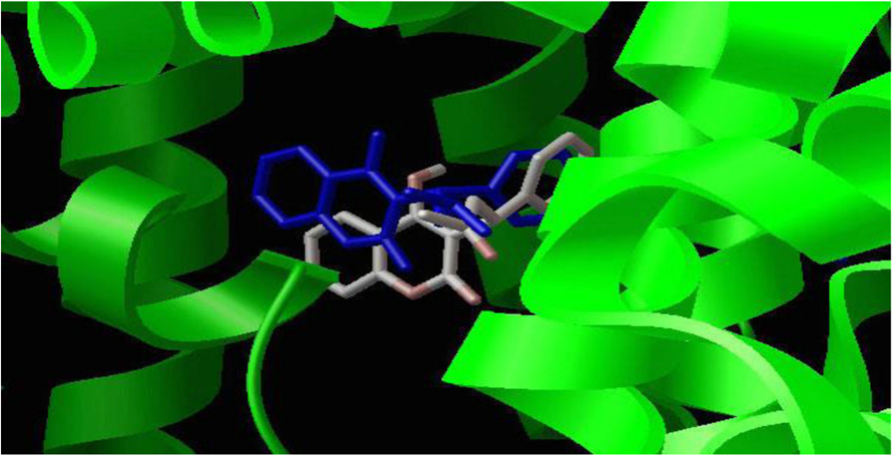
**The docking conformation and location of warfarin on the Human serum albumin (PDB ID: 1H9Z).** The conformation of warfarin after docking indicates with DG colored sketch (David Goodsell’s scheme) and what was retrieved from the crystallographic study indicate as a blue sketch.

First of all, in this process, the accuracy of applied docking protocol was examined. So, warfarin as a probe of HSA (whose high affinity binding site was known as a subdomain IIA) was docked into HSA (PDB ID: 1H9Z), which co-crystallized with it. Docking results demonstrates that the warfarin docks back into the experimental binding location which was retrieved based on the crystallographic study (Figure 6).Therefore; this protocol was confirmed and consequently, applied in docking simulation for complexes of quercetin with HSA and BSA.

**Figure 7:**
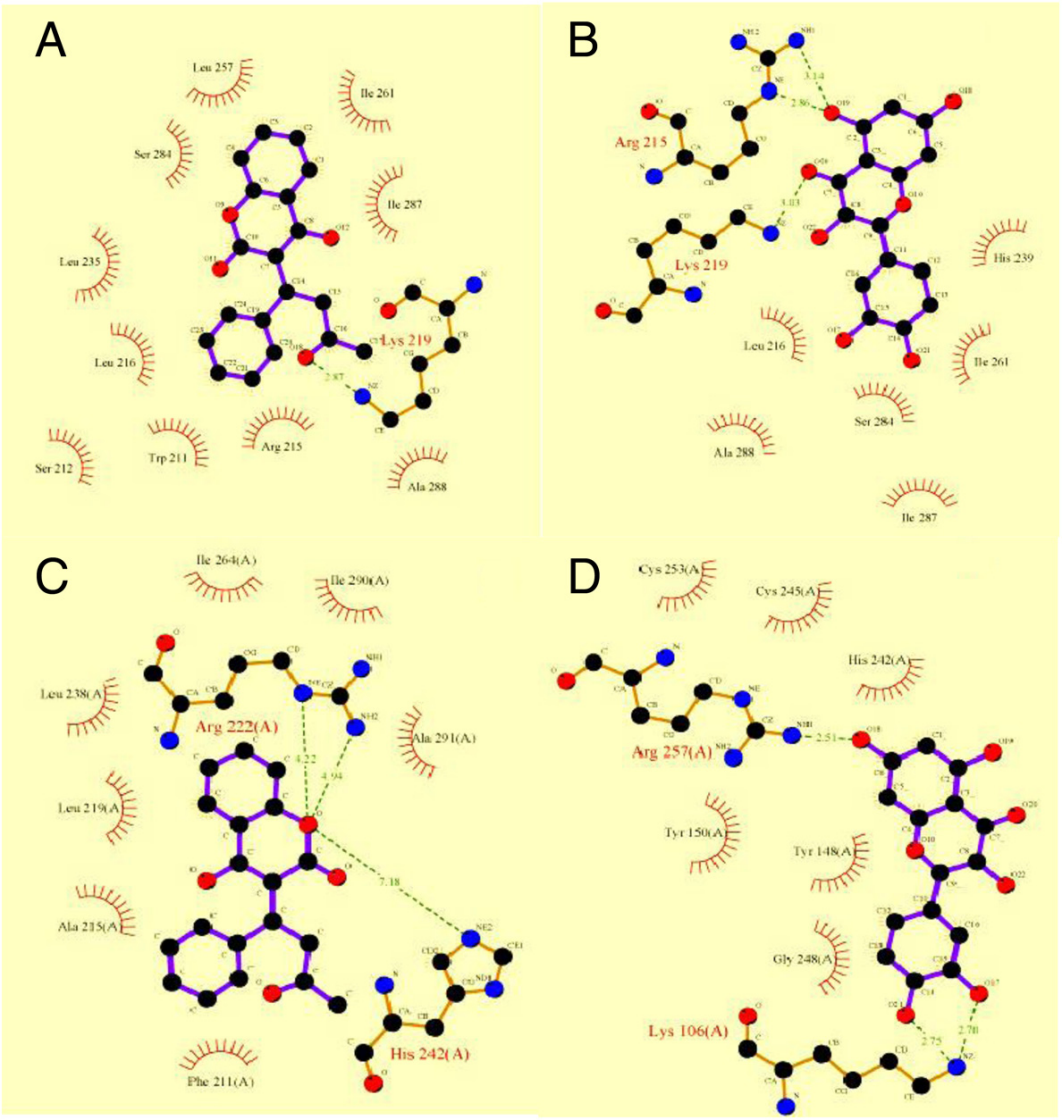
**The structure of bovine serum albumin complexes with warfarin and quercetin (A, B), consequently (C) The structure of human serum albumin complexes with warfarin and quercetin, (D) was analyzed using the Ligplot 4.22 program.** The hydrogen bonds are shown as dash lines and hydrophobic contacts are indicated with half-moon.

For analyzing the specific contact and indicating the participated amino acids in biding sites the Ligplot program was employed [44]. It is indicated in Figure 7, That in the binding site of bovine serum albumin, serine 284, isoleucine 261 and 287, alanine 288 involve in hydrophobic interaction with quercetin and warfarin, and also arginine 219 contribute in a hydrogen bond with both, this means that in the case of BSA, the warfarin and quercetin binding sites completely overlap, while these ligands are bonded in different location in HSA.

The Dufour and Dangles fluorescence spectroscopy study had shown that fluorescence of BSA-quercetin complex (protein/marker molar ratio of 1) is affected by constant concentration of warfarin (site marker of subdomain IIA ), but fluorescence of HSA-quercetin complex is independent of warfarin presence [33].

For better understanding of the relation between experimental observations and what was retrieved from docking calculations. The more investigations were taken to visualization the effect of BSA-flavonoids interaction on local conformational changes, which leads to fluorescence quenching.

First notable issue which was considered is that the amino acids lining in the common binding site of quercetin and warfarin, are neighbors of tryptophan 212 within 5Ǻ. This is important because the intrinsic fluorescence of BSA with excitation at 295 nm is due to the tryptophan residues (BSA has two tryptophan, tryptophan 212 and tryptophan 134) and quercetin actually promoted a strong quenching of the tryptophan emission at 340 nm [33]. These observations lead to further study of checking the tryptophan flexibility after interaction between the quercetin and BSA complex.

**Figure 8:**
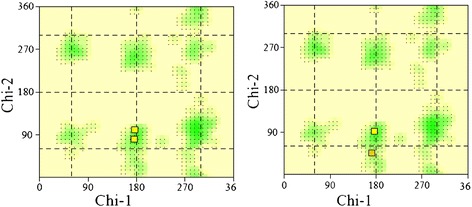
**The χ**_**1**_ **/χ**_**2**_ **plots are obtained from PROCHECK program.** Each square represents one residue. The color coding of the squares is corresponding the range of yellow to red which is reflects the degree to which the orientation is favored; therefore, yellow indicate the residue is in a favored and red indicate the residue is an unflavored region. Two tryptophan of bovine serum albumin was indicated in both plots. The left plot indicate χ_1_/χ_2_ coordinate of tryptophans before MD (Left). The right plot indicates the χ_1_/χ_2_ coordinates of tryptophans after MD (Right). How it is shown, trp 134 remains constant while the χ_1_/χ_2_ coordinates of trp 212 is changed and located in unflavored region.

So, the complex of BSA model and quercetin (best conformation after docking) was subjected to molecular dynamic simulation for 2 ns. After the stability of the system, for describing the side chain conformational changes the dihedral angles of our interested aromatic residue, χ_1_ / χ_2_ plot was provided by PROCHECK program [31]. The results represent the significant shuffling between side-chain conformations of tryptophan 212 before and after the refinement of BSA-quercetin complex with molecular dynamic simulation. Figure 8 indicates that the BSA residues which were involved in interaction with quercetin, pushing the tryptophan 212 side chain conformations to unflavored regions.

## Discussion

In present work, based on docking calculation and molecular dynamic simulation, the detailed understanding was obtained about the interaction of quercetin (as a typical flavonoids) and BSA, which is in consistence with previous experimental data [33]. In addition, these calculations in molecular levels demonstrate its ability to providing the interpretation for experimental observation.

The first-stage docking results confirm on one hand the reliability of obtained 3D structural model, and on the other hand, by showing the significant specificity between high homologous BSA and HSA proteins, indicate the necessity of constructing BSA model’s for analyzing the interaction flavonoids with it.

According to visualization of the predicted binding site of quercetin, any interventions are not observed between the quercetin binding site and reported glycation site of BSA [18]. However, for the more reliable conclusion, a number of flavonoids which introduced as an inhibitor of BSA glycation [3,12] were employed for the further docking studies.

By the speculation that the radical scavenging activity in physiological condition could inhibit oxidative fragmentation of Amadori rearrangement product and subsequently prevented the AGEs formation, the number of flavonoids was studied to find a correlation between the experimental anti glycation activity and radical scavenging activity of them [12]. In the present investigation with respect to their works, interested flavonoids was selected and by considering an interference reaction in the glycation site as the possible inhibitory mechanism [9,45,46], the theoretical binding energies of flavonoids in the complex with BSA were calculated by docking methods(calculated free energy of binding were listed in Tables 3 and 4). Then, analytical methods were performed by SPSS software (version 15). The correlation analyzes between the experimental activity (IC_50_) and predicted binding energy reveal that there is no significant correlation between these values (p > 0.3).

**Table 3:** **The free energy of binding between, 12 Flavones, 12 Flavonols, 3 Flavanones, 2 Flavan-3-ols and bovine serum albumin obtained from docking study, and their AGEs inhibitions’ IC**_**50**_ **(in regard to Matsuda, H., et al.** [12]**)**


**Num**	**Name**	**R** ^**1**^	**R** ^**2**^	**R** ^**3**^	**R** ^**4**^	**R** ^**5**^	**R** ^**6**^	**R** ^**7**^	**R** ^**8**^	**ΔG (Kcal/mol)**	**AGEs inhibition IC**_**50**_ **(% in 200 μM)**
**1**	Flavone	H	H	H	H	H	H	H	H	−7.09	5%
**2**	7-Hydroxyflavone	H	H	OH	H	H	H	H	H	−6.96	27%
**3**	Chrysin	OH	H	OH	H	H	H	H	H	−7.56	43%
**4**	4,7-Dihydroxyflavone	H	H	OH	H	H	OH	H	H	−7.0	44%
**5**	3,4,7-Trihydroxyflavone	H	H	OH	H	OH	OH	H	H	−7.53	44%
**6**	Apigenin	OH	H	OH	H	H	OH	H	H	−7.52	53%
**7**	Luteolin	OH	H	OH	H	OH	OH	H	H	−7.53	64%
**8**	Diosmetin	OH	H	OH	H	OH	OCH_3_	H	H	−7.05	27%
**9**	Pilloin	OH	H	OCH_3_	H	OH	OCH_3_	H	H	−7.9	29%
**10**	10 F	OH	H	OCH_3_	H	OCH_3_	OCH_3_	H	H	−7.0	29%
**11**	Wogonin	OH	H	OH	OCH_3_	H	H	H	H	−7.03	50%
**12**	Baicalin	OH	OH	OH	H	H	H	H	H	−7.75	79%
**13**	3-Hydroxyflavone	H	H	H	H	H	H	H	OH	−7.1	3%
**14**	Kaempferol	OH	H	OH	H	H	OH	H	OH	−7.47	46%
**15**	Quercetin	OH	H	OH	H	OH	OH	H	OH	−7.67	57%
**16**	Rhamnetin	OH	H	OCH_3_	H	OH	OH	H	OH	−7.57	55%
**17**	Tamarixetin	OH	H	OH	H	OH	OCH_3_	H	OH	−7.89	45%
**18**	F18	OH	H	OCH_3_	H	OH	OH	H	OCH_3_	−7.4	57%
**19**	Ombuine	OH	H	OCH_3_	H	OH	OCH_3_	H	OH	−7.66	27%
**20**	Ayanin	OH	H	OCH_3_	H	OH	OCH_3_	H	OCH_3_	−7.66	40%
**21**	21 F	OH	H	OCH_3_	H	OCH_3_	OCH_3_	H	OH	−7.69	13%
**22**	Myricetin	H	H	H	H	H	H	H	OH	−7.48	61%
**23**	Mearnsetin	H	H	H	H	H	CH3	H	OH	−7.51	10%
**24**	24 F	H	H	CH_3_	H	H	H	H	OH	- 7.81	42%
**25**	Liquiritigenin	H	H	OH	H	H	OH	H	H	−7.16	40%
**26**	26 F	H	H	OCH_3_	H	H	OH	H	H	−6.74	44%
**27**	Eriodictyol	OH	H	OH	H	OH	OH	H	H	−7.46	46%
**28**	(+)-Catechin	OH	H	OH	H	OH	OH	H	β-OH	−7.74	69%
**29**	(−)-Epigallocatechin	OH	H	OH	H	OH	OH	OH	α-OH	−7.06	4%

**Table 4:** **The free energy of binding between 3 isoflavones and bovine Serum albumin obtained from docking study, and their AGEs inhibitions’ IC**_**50**_


**Num.**	**Name**	**R** ^**1**^	**R** ^**2**^	**R** ^**3**^	**R** ^**4**^	**R** ^**5**^	**ΔG (Kcal/mol)**	**AGEs inhibition IC**_**50**_ **(% in 200 μM)**
**30**	Daidzein	H	H	OH	H	OH	−7.05	33%
**31**	Genistein	OH	H	OH	H	OH	−6.44	34%
**32**	Biochanin A	OH	H	OH	H	OCH_3_	−7.68	36%

**Figure 9:**
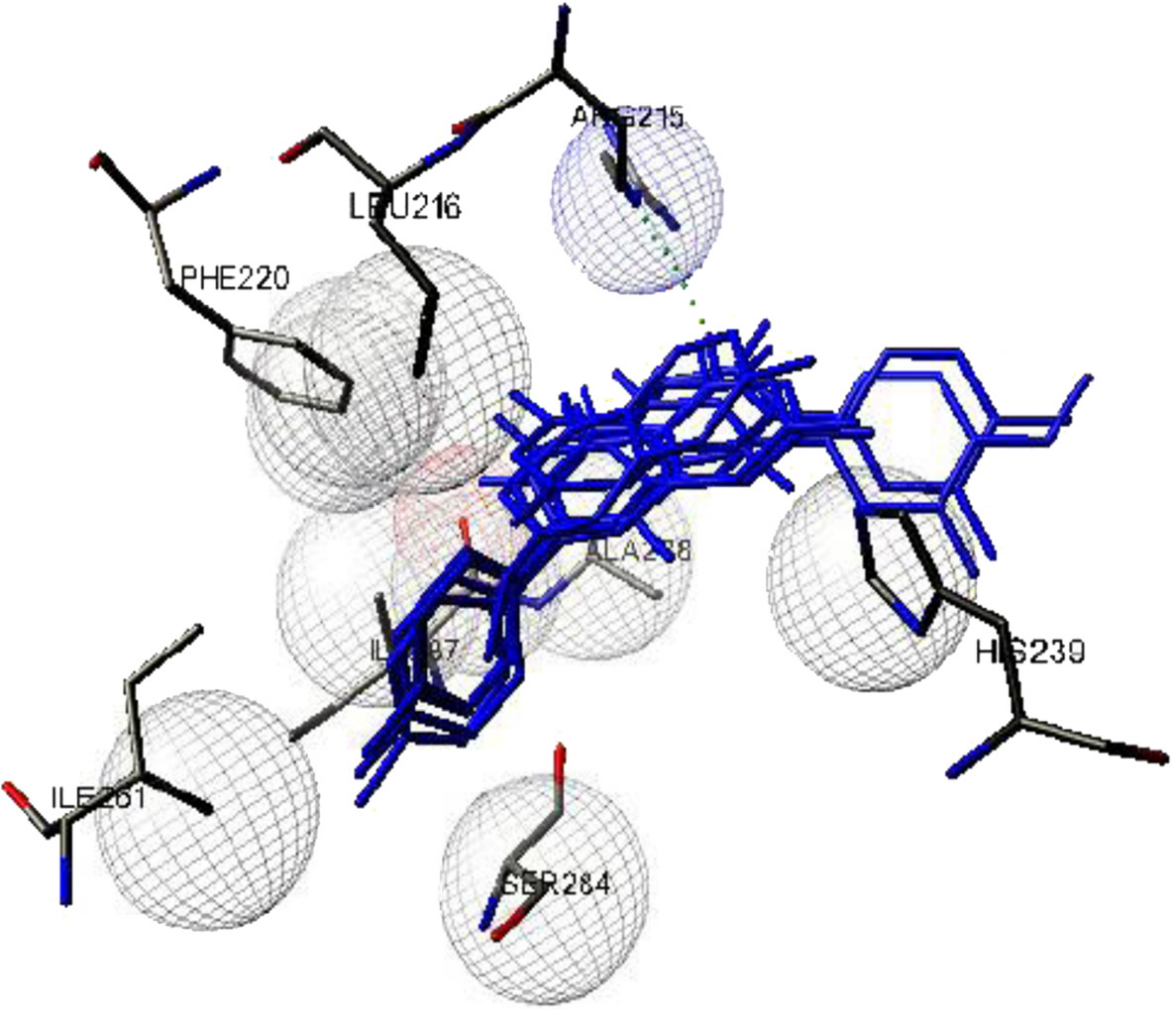
**The superimposition of docked complex.** The most likely conformation of representative flavonoids of under study groups (Flavones, flavonols, flavanones, flavan-3-ols, isoflavones), is represented in their binding site.

Besides the statistical analyzes, the AutoDockTools (ADT) was used for visualization the relationship between the predicted flavonoids’ binding site and the reported glycation site on BSA. Interestingly, despite the structural differentiations which exist among these flavonoids, the most likely conformation of each one of them, were placed in the same binding site (consist of lysine 219, arginine 215, isoleucine 287, and serine 284) (Figure 9) which can’t be considered as any glycation sites of BSA (in respect to aforementioned Hinton and Ames study). Therefore, by considering these results, maybe it can be concluded that the hydrophobic hindrances in the glycation site can’t be considered as an inhibitory mechanism for flavonoids.

## Conclusion

In previous experimental studies, the high homology between BSA and HSA has invoked speculation that they have a similar structure and so share similar capability in some of their properties like ligands binding [45]. However, simulating the quercetin (as the most studied flavonoids) interactions with both BSA and HSA show significant differences between them, which confirms our speculation about the necessity of providing the affordable BSA 3D structural model for analyzing its specific binding location and binding affinity for our interested ligands.

Interestingly, in the present study, the consistency between docking results and experimental data not only indicate the validity of our simulation but also become helpful in interpreting about experimental observation in molecular levels [33].

Thus different aspect of results was considered, on one hand the visualization of quercetin binding site leads to find the relationship between the binding site residues and tryptophan 212, and also by employing the MD simulation it is revealed that participated residues in flavonoids interaction site affect on tryptophan local conformational change, which at last is observed as fluorescence quenching.

On the other hand, according to our obtained results, the possibility of relation between the flavonoids binding site and glycation sites was investigated, while the correlation analyzes was adopted between binding affinity of flavonoids to BSA and IC_50_ of AGE inhibition. How was mentioned before based on the results, the flavonoids can’t be considered as an intervention agent for glycation site and therefore, the possibility of this inhibitory mechanism is unacceptable in the case of flavonoids.

**Figure 10:**
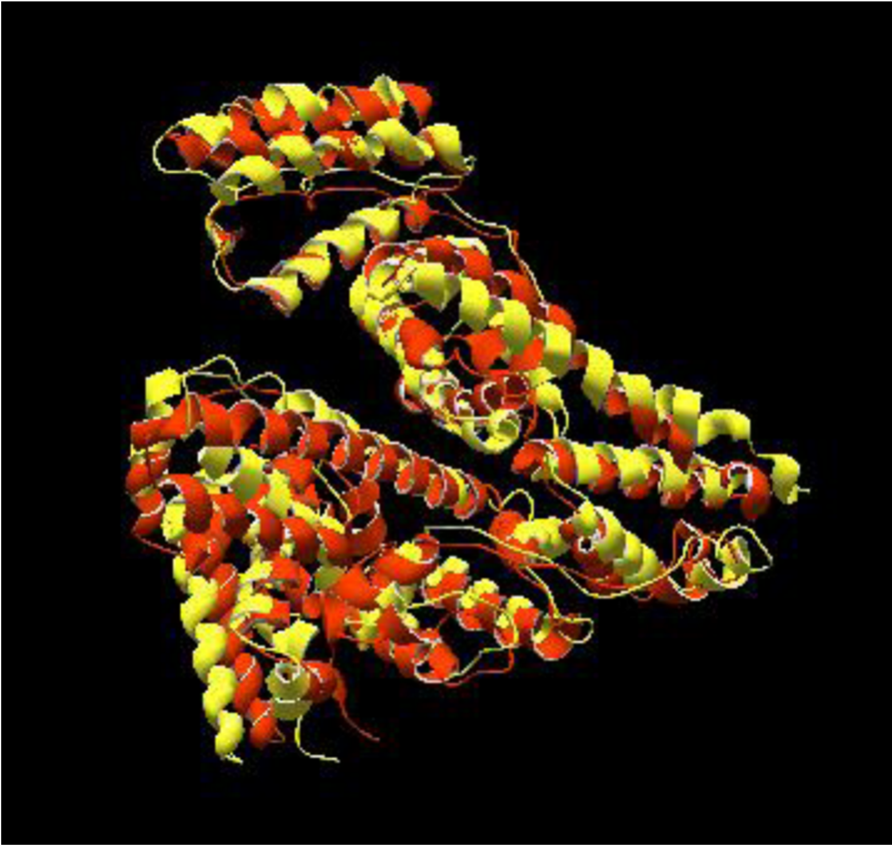
**The superimposition between crystallographic structure of bovine serum albumin (PDB ID: 4F5S) (orange) and calculated 3D structure model (yellow), obtained with Swiss PDB viewer software.**

Ultimately, the other interesting outcome of this study is laid beneath the final examination. Coincident with our study the crystallographic structure of bovine serum albumin was determined [15]. Therefore, the comparison has been made between our calculated 3D structural models of BSA with the reported model which retrieved from crystallographic technique to get insight into their distinctive difference. These two structures were superimposed with Swiss PDB viewer software (Figure 10) [47]. And strong correlation (RMSD: 1.9) between them shades a light in the hypothesis that applied methods in this study are accurate and reliable.
